# Phenotype and outcomes of very early onset and early onset inflammatory bowel diseases in a Montreal pediatric cohort

**DOI:** 10.3389/fped.2023.1157025

**Published:** 2023-04-04

**Authors:** Laurence Chapuy, Bertrand Leduc, David Godin, Amélie Damphousse, Nathalie Patey, Dorothee Dal Soglio, Prevost Jantchou, Colette Deslandres

**Affiliations:** ^1^ Division of Pediatric Gastroenterology, Hepatology and Nutrition, Department of Pediatrics, CHU Sainte-Justine, University of Montreal, Montreal, QC, Canada; ^2^ CHU Sainte Justine Research Center, University of Montreal, Montréal, QC, Canada; ^3^ CRCHUM, University of Montreal, Montreal, QC, Canada; ^4^ Faculty of Medicine, McGill University, Montreal, QC, Canada; ^5^ Department of Radiology, CHU Sainte-Justine, University of Montreal, Montreal, QC, Canada; ^6^ Department of Pathology, CHU Sainte-Justine, University of Montreal, Montreal, QC, Canada

**Keywords:** phenotype, outcomes—health care, early-onset IBD, very early onset IBD, mucosal inflammation, sustained remission, magnetic resonance enterography

## Abstract

**Objectives:**

The incidence of very-early-onset inflammatory bowel disease (VEO-IBD) and early-onset IBD (EO-IBD) is increasing. Here, we report their phenotype and outcomes in a Montreal pediatric cohort.

**Methods:**

We analyzed data from patients diagnosed with IBD between January 2014 and December 2018 from the CHU Sainte-Justine. The primary endpoint was to compare the phenotypes of VEO-IBD and EO-IBD. The secondary endpoints involved comparing outcomes and rates of steroid-free clinical remission (SFCR) at 12 (±2) months (m) post-diagnosis and at last follow-up.

**Results:**

28 (14 males) and 67 (34 males) patients were diagnosed with VEO-IBD and EO-IBD, respectively. Crohn's disease (CD) was more prevalent in EO-IBD (64.2% vs. 39.3%), whereas unclassified colitis (IBD-U) was diagnosed in 28.6% of VEO-IBD vs. 10.4% of EO-IBD (*p* < 0.03). Ulcerative colitis (UC) and IBD-U predominantly presented as pancolitis in both groups (VEO-IBD: 76.5% vs. EO-IBD: 70.8%). Combining all disease subtypes, histological upper GI lesions were found in 57.2% of VEO-IBD vs. 83.6% of EO-IBD (*p* < 0.009). In each subtype, no differential histological signature (activity, eosinophils, apoptotic bodies, granulomas) was observed between both groups. At 12 m post-diagnosis, 60.8% of VEO-IBD and 62.7% of EO-IBD patients were in SFCR. At a median follow-up of 56 m, SFCR was observed in 85.7% of VEO-IBD vs. 85.0% of EO-IBD patients.

**Conclusion:**

The rate of patients in SFCR at 1-year post-diagnosis and at the end of follow-up did not significantly differ between both groups.

## Introduction

Inflammatory bowel diseases (IBD), including Crohn's disease (CD), ulcerative colitis (UC), and unclassified colitis (IBD-U), result from an abnormal immune response to environmental factors, including the gut microbiota, in genetically predisposed subjects (1). IBD are polygenic diseases with more than 230 risk loci reported in genome-wide association studies (1). Hence, environmental factors are key players in the pathophysiology of IBD, especially in adults and teenagers. Although the incidence of pediatric IBD is highest during adolescence (2, 3), IBD can also affect younger patients, defining early onset-IBD (EO-IBD) in children diagnosed from the age of 6–10 years and very early onset-IBD (VEO-IBD) in patients diagnosed before the age of 6 years (4). In VEO-IBD patients, genetic contribution in the pathophysiology of the disease is stronger, with numerous rare monogenic disorders reported over the last 10 years (5, 6). Nonetheless, identification of specific mutations associated with VEOIBD is observed in only 3% of a pediatric cohort (0–18 years), with an overrepresentation under 2 years old (7). Therefore, monogenic IBD represents only 7.8% of VEO-IBD (7).

Thus, most cases of VEO-IBD are not associated with the identification of monogenic disorders and are underreported in the literature compared to monogenic IBD. Only a few cohorts have described the phenotype at diagnosis and treatments of VEO-IBD (4, 8–13). Detailed histological and radiologic findings are even more sparsely reported in the literature (14, 15).

We hypothesized that the clinical phenotype, histological and radiologic findings, as well as treatment and outcomes of VEO-IBD are different from those observed in EO-IBD. Therefore, the primary study aim was to compare the baseline characteristics and longitudinal outcomes of patients diagnosed with VEO-IBD and EO-IBD at our tertiary center between January 2014 and December 2018. The secondary aims were to investigate the response to treatments and sustained remission during follow-up between the two cohorts of VEO-IBD and EO-IBD.

## Methods

### Study design, patients' cohort, data collection

This was a retrospective cohort study conducted at a single tertiary medical center (Sainte-Justine University Hospital Center, Montreal, Canada), including all the patients diagnosed with IBD. Patients were selected from a prospective IBD database maintained in the Department of Pediatric Gastroenterology at CHU Sainte-Justine (16), and data were collected from clinical charts.

The inclusion criteria were (1) age between 0 and less than 10 years at the time of diagnosis, (2) IBD diagnosed between January 2014 and December 2018, (3) patients followed at CHU Sainte-Justine at any time between January 2014 and December 2018. The diagnosis of IBD has been made using validated clinical, endoscopic, histological, and radiologic criteria. To ensure an accurate diagnosis classification of CD, UC, and IBD-U, diagnosis criteria have been reviewed by two blinded independent researchers (LC; CD), using the revised Porto criteria (17) and the Pediatric-IBD classes (18).

Patients were divided into two cohorts: (1) VEO-IBD, defined as an age strictly inferior to 6-year-old at diagnosis, and (2) EO-IBD, defined as an age between 6 and inferior to 10-year-old at diagnosis. Data were collected at diagnosis, at 1-year post-diagnosis (±2 months), and at the last visit before the end of follow-up at the latest in December 2021.

We collected the following variables: (1) clinical variables: patient's sex, age, weight, height, BMI *z*-scores, symptoms as defined in the Paris classification (19), disease activity, Physician Global Assessment (PGA) at diagnosis, at 12 months and at last follow-up; (2) endoscopic variables at diagnosis; (3) histological variables at diagnosis; (4) immunological data (neutrophil oxidation test, post-immunization serologies, lymphocyte subset count and immunoglobulin subclass levels) at diagnosis; (5) radiologic variables at diagnosis; (6) genetic data; (7) induction (treatment used between 0 and 2 months from diagnosis) and maintenance treatments; (8) clinical disease activity during follow-up at 12 months and at last follow-up; (9) weight, height, BMI *z*-scores, fecal calprotectin (ELISA method: immundiagnostik® IDK kit), and endoscopic data at the end of follow-up when available. Fecal calprotectin and endoscopic data were collected at the end of follow-up if available in a period of ±2 months from the last follow-up visit.

Sustained corticosteroid free clinical remission (SFCR) was defined by a short pediatric Crohn's disease activity index (sPCDAI) or a pediatric ulcerative colitis activity index (PUCAI) lower than 10.

Histological data were analyzed from biopsies collected during endoscopy. For all patients, at least one biopsy was taken from each of the following segments: rectum, sigmoid colon, descending colon, transverse colon, ascending colon, and ileum when accessible. For upper gastrointestinal endoscopy, at least one biopsy was taken from the esophagus, stomach body and antrum, and duodenum. Data collected included variables associated with (1) chronic intestinal inflammation (lympho-plasmocytic infiltrate, architectural disarray, basal plasmocytosis) and (2) disease activity according to the intensity of the neutrophilic infiltrate [quoted as mild (neutrophils limited to lamina propria or isolated cryptitis), moderate (cryptitis with crypt abscesses) or severe (diffuse active inflammation with ulceration or granulation tissue)]. The presence and location of granulomas were also collected, as were apoptotic bodies and eosinophils. Eosinophil infiltration was defined as followed: (0) absent; (1) present within normal limits in the lamina propria; (2) increased in lamina propria (>1/hpf in the esophagus; >5/hpf in the stomach; >15/hpf in the duodenum; >18/hpf in the ileum; >29/hpf in the caecum; >22/hpf in the transverse colon; and >14/hpf in the sigmoid colon); (3) increased in lamina propria as above with eosinophil crypt abscesses; (4) increased in lamina propria as above and involving surface epithelium (14).

Histological data were analyzed from all biopsies collected during endoscopy. A unique pathologist (DD) reviewed all the slides to ensure the comparability of the data.

Radiologic procedures, including magnetic resonance enterography (MRE) (20), small bowel follow-through (SBFT), and abdominal ultrasound (abdo-US) were analyzed. We analyzed the average interval between the date of diagnosis and the use of an abdo-US or a MRE. On MRE, we defined small bowel lesions as followed: short segment involvement: <15 cm; medium segment involvement: 16–30 cm; long segment involvement: >30 cm. The IBD colonic extension on MRE was not evaluated in this study. A review of radiology reports from pediatric radiologists was used to extract radiologic data in the study.

### Data analysis

Data were analyzed as a comparison of VEO-IBD and EO-IBD cohorts. The categorial variables were analyzed by the chi-square method. The continuous variables were analyzed by Student's *t*-tests or Wilcoxon non-parametric test. Statistical significance was defined as *p*-value <0.05. Analyses were performed using GraphPad Prism® version 7.

## Results

### Cohort characteristics at diagnosis

Between January 2014 and December 2018, 549 newly diagnosed IBD patients were identified in our pediatric IBD database. Twenty-eight patients under 6 years old (VEO-IBD) and 67 patients aged from 6 to 10 years (EO-IBD) at diagnosis fulfilled the inclusion criteria, accounting for 5.1% and 12.2% of the cohort, respectively (Table 1). Monogenic forms of IBD were not included in our database. Twenty-six (93%) of VEO-IBD patients, including five patients less than 2 years old at diagnosis, underwent an immune workup at diagnosis that was not suggestive of an underlying immune deficiency. Genetic testing are summarised in Supplementary Table S1.

The proportion of patients with IBD-U was higher in the VEO-IBD vs. EO-IBD group whereas CD was overrepresented in the EO-IBD group (*p* < 0.03). Isolated colonic disease (L2 phenotype) was found in 81.9% of VEO-CD patients, as compared to only 25.6% of EO-CD patients (*p* < 0.004). Likewise, most patients in the EO-CD group had a L3 phenotype (53.5%). L4 phenotype was overrepresented in the EO-CD group, with 81.4% of patients displaying lesions in the upper gastrointestinal tract compared to 54.5% in the VEO-CD group (*p* < 0.004). A perianal disease was found in 36.4% and 20.9% of VEO-CD and EO-CD patients, respectively (*p *= 0.42). In both groups, more than 70% of UC and IBD-U patients presented with a pancolitis at diagnosis. One patient presented with psoriasis in each group, and two had arthritis in the EO-IBD group. Three patients (2 UC and 1 IBD-U) had concurrent sclerosing/autoimmune cholangitis in the EO-IBD group. No patients presented with ophthalmic diseases. The median body mass index (BMI) *z*-score was not significantly different between the VEO-IBD [−0.14 (IQR = −0.96 to 0.6)] and the EO-IBD cohort [−0.59 (IQR = −1.47 to 0.25)] (Table 1). However, when both cohorts were pooled according to diagnosis, CD patients had a lower BMI *z*-score [−0.86 (−1.6 to 0.17)] than UC/IBD-U patients [−0.06 (−0.85 to 0.39)] (*p* < 0.05).

**Table 1:** Patient demographics and clinical characteristics at diagnosis.

	VEO-IBD				EO-IBD			
	Total	CD	UC	IBD-U	Total	CD	UC	IBD-U
**Demographics**								
*N* (% total cohort)	28	11 (39.3)*	9 (32.1)	8 (28.6)*	67	43 (64.2)*	17 (25.4)	7 (10.4)*
Females, *n* (%)	14 (50)	5 (45)	5 (56)	4 (50)	33 (49.3)	18 (42)	11 (64.7)	4 (57.1)
Age, median (range)	4.3 (0.8–5.9)	4.2 (0.8–5.9)	4.9 (1.6–5.9)	3.1 (0.7–5.1)	8.7 (6–9.8)	8.4 (6–9.8)	8.8 (6.9–9.7)	8.7 (6–9)
**Symptoms**								
Diarrhea, *n* (%)	21 (75)	8 (72.7)	6 (66.7)	7 (87.5)	43 (64.2)	26 (60)	11 (64.7)	6 (85.7)
Blood in stools, *n* (%)	26 (92.8)	9 (81.8)	9 (100)	8 (100)	42 (62.7)	19 (44)	16 (94)	7 (100)
Abdominal pain, *n* (%)	16 (57.1)	8 (72.7)	7 (77.8)	1 (12.5)	54 (80.5)	38 (88)	10 (58.8)	6 (85.7)
EI manifestations, *n* (%)	1 (3.6)	0	1 (11.1)	0	6 (9.0)	3 (9.3)	2 (11.8)	1 (14.3)
**Disease location—UC**								
Proctitis—E1			0	0			3 (17.6)	0
Left side colitis—E2			1 (11.1)	2 (25)			0	1 (14.3)
Extensive colitis—E3			1 (11.1)	0			1 (5.9)	1 (14.3)
Pancolitis—E4			7 (77.8)	6 (75)			12 (70.6)	5 (71.4)
N/A							1 (5.9)	
**Disease location—CD**								
Terminal ileum—L1		0 (0)				7 (16.3)		
Colon only—L2		9 (81.8)**				11 (25.6)**		
Ileocolonic—L3		2 (18.2)				23 (53.5%)		
Upper GI tract only—pure L4		0				2 (4.7)		
Upper GI tract—L4		6 (54.5)**				35 (81.4)**		
L4a		6 (54.5)				27 (62.8)		
L4b		0				2 (4.7)		
L4ab		0				6 (14.0)		
**Disease behavior—CD**								
Non strict./Non penet.		11 (100)				36 (83.7)		
Stricturing		0				5 (11.6)		
Penetrating		0				2 (4.7)		
Perianal disease		4 (36.4)				9 (20.9)		
**PGA**								
Mild	4 (14.3)	2	1	1	12 (17.9)	8	2	2
Moderate	7 (25)	2	3	2	26 (38.8)	15	8	3
Severe	17 (60.7)	7	5	5	29 (43.3)	20	7	2
**BMI *z*-score** ^a^	−0.14	−0.78	0.24		−0.59	−0.96	−0.3	
[IQR]	[−0.96/0.6]	[−1.1/1.28]	[−0.45/0.54]		[−1.47/0.25]	[−1.7/0.13]	[−0.9/0.3]	

^a^ Median body mass index (BMI) *z*-score was not significantly different between the VEO-IBD and the EO-IBD cohort. However, when both cohorts were pooled according to diagnosis, CD patients had a lower BMI *z*-score [−0.86 (−1.6 to 0.17)] than UC/IBD-U patients [−0.06 (−0.85 to 0.39)] (*p *< 0.05).

* *p* < 0.05.

** *p* < 0.01.

### Histological data

In Crohn's disease, the frequency of microscopic gastritis was higher in the EO-CD (88.4%) vs. VEO-CD group (63.6%) (*p* < 0.05) (Table 2). Whereas all patients in the VEO-CD group had chronic colitis, 16.3% of EO-CD patients were diagnosed with CD in absence of chronic inflammation in colon or ileum. Granulomas were found in equal proportion in both CD groups. Increased eosinophils were found in 15 out of 43 (34.8%) EO-IBD patients and 3 out of 11 (27.2%) VEO-IBD patients.

**Table 2:** Pathology at diagnosis.

	CD		UC and IBD-U	
	VEO	EO	VEO	EO
	(*n* = 11%)	(*n* = 43%)	(*n* = 17%)	(*n* = 24%)
**Upper GI endoscopy**				
Normal (macroscopy and histology)	4 (36.4)	5 (11.6)	8 (47.1)	6 (25)
Isolated chronic gastritis	2 (18.2)	15 (34.9)	9 (52.9)	13 (54.2)
Chronic gastritis and esophagitis	0	5 (11.6)	0	0
Chronic gastritis and duodenitis	3 (27.3)	5 (11.6)	0	0
Chronic gastritis, esophagitis and duodenitis	2 (18.2)	9 (20.9)	0	0
Isolated duodenitis	0	1 (2.3)	0	0
Duodenitis and esophagitis	0	3 (7.0)	0	0
Helicobacter Pylori gastritis	0	0	0	1 (4.2)
N/A	0	0	0	4 (16.7)
**Lower GI endoscopy**				
Chronic colitis without ileitis	6 (54.5)	13 (30.2)	17 (100)	24 (100)
No or mild activity	6	5	6 (35.3)	6 (25)
Moderate activity	0	6	7 (41.2)	14 (58.3)
Severe activity	0	2	4 (23.5)	4 (16.7)
Eosinophils	2	4	4 (23.5)	3 (12.5)
Apoptotic bodies	0	2	0	2 (8.4)
Chronic colitis and ileitis	2 (18.2)	17 (39.5)		
No or mild activity	1	11		
Moderate activity	0	5		
Severe activity	1	1		
Eosinophils	0	10		
Apoptotic bodies	0	2		
Chronic colitis with no data available for ileum	3 (27.3)	4 (9.3)		
No or mild activity	1	3		
Moderate activity	1	1		
Severe activity	1	0		
Eosinophils	1	1		
Apoptotic bodies	1	2		
Chronic ileitis	0	2 (4.6)		
Eosinophils	0	0		
Apoptotic bodies	0	1		
No inflammation in colon or terminal ileum	0	7 (16.3)		
**Granulomas**	7 (63.6)	29 (67.4)		

In UC and IBD-U patients, 17 out of 24 (70.8%) EO and 11 out of 17 (64.7%) VEO patients had a macroscopically normal upper GI endoscopy. However, more than 50% of both groups had a chronic gastritis upon histopathological examination. Colonic chronic inflammation was found in the whole UC and IBD-U cohort in both groups. Eosinophils were found in 3 out of 24 (12.5%) EO-IBD patients and 4 out of 17 (23.5%) VEO-IBD patients (Table 2).

### Radiologic data

Almost all VEO-IBD patients had at least one abdo-US (96.3% of 27 patients) compared to three quarters (74.6%) in the EO-IBD patients. The use of small bowel follow-through was more prevalent in the VEO-IBD (33.3%) than the EO-IBD patients (6.0%). In contrast, MRE was more frequently performed in the EO-IBD (92.5%) vs. VEO-IBD patients (33.3%). Therefore, radiologic data showed a shift from the abdo-US to MRE imaging as the age of the patients increased. The average interval between the date of diagnosis and MRE was shorter in the VEO-IBD than EO-IBD group (7.3 vs. 34.7 days in the VEO-IBD vs. EO-IBD group, respectively). In concordance with endoscopic findings, ileal lesions on MRE were more prevalent in EO-IBD than VEO-IBD patients (Supplementary Table S2).

### Treatment and outcomes

Induction treatments used in both cohorts are summarized in Table 3. Overall, the use of corticosteroids to induce remission was more common in VEO-IBD than EO-IBD patients regardless of disease subtypes [VEO-CD: *n *= 7 (63.6%) vs. EO-CD: *n *= 20 (46.5%)—VEO-UC/IBD-U: *n *= 15 (88.2%) vs. EO-UC/IBD-U: *n *= 12 (50%) (*p* < 0.05)]. During the first 2 months after diagnosis, TNFα-inhibitor therapies were used in about 35% of patients [EO-CD: *n *= 17 (39.5%)—VEO-UC/IBD-U: *n *= 6 (35.3%)—EO-UC/IBD-U *n *= 8 (33.3%)], except in VEO-CD (27.3%).

**Table 3:** Induction treatment.

	CD		UC and IBD-U	
	VEO	EO	VEO	EO
	*n *= 11 (%)	*n *= 43 (%)	*n *= 17 (%)	*n *= 24 (%)
Exclusive Enteral Nutrition alone	1 (9.1)	6 (14.0)	0	0
Corticosteroids alone	2 (18.2)	7 (16.3)	4 (23.5)	5 (20.8)
Corticosteroids + Sulfasalazine	0	0	3 (17.6)	0
Corticosteroids + Mesalamine	2 (18.2)	4 (9.3)	2 (11.7)	4 (16.7)
Corticosteroids + Adjuvant Exclusive Enteral Nutrition	0	2 (4.6)	0	0
Corticosteroids + Anti-TNF-α	3 (27.3)	6 (14.0)	6 (35.3)	3 (12.5)
Anti-TNF-α alone	0	5 (11.6)	0	3 (12.5)
Anti-TNF-α + Mesalamine	0	1 (2.3)	0	2 (8.3)
Anti-TNF-α + Adjuvant Exclusive Enteral Nutrition	0	4 (9.3)	0	0
Corticosteroids + anti-TNF-α + Adjuvant Exclusive Enteral Nutrition	0	1 (2.3)	0	0
Sulfasalazine alone	1 (9.1)	0	1 (5.9)	1 (4.2)
Mesalamine alone	2 (18.2)	6 (14.0)	0	6 (25)
Colchicine alone	0	1 (2.3)	0	0
N/A	0	0	1 (5.9)	0
Total Exclusive Enteral Nutrition	1 (9.1)	15 (34.9)	0	0
Total Corticosteroids	7 (63.6)	20 (46.5)	15 (88.2)	12 (50)
Total anti-TNF-α	3 (27.3)	17 (39.5)	6 (35.3)	8 (33.3)

The median follow-up was 53 months (range 25–88 months) and 59 months (range 29–90 months) for VEO-IBD and EO-IBD cohorts, respectively. At 1-year (±2 months) after diagnosis, patients in steroid-free clinical remission (SFCR) were equally distributed in all cohorts (VEO-CD 63.6% vs. EO-CD 65.1% and VEO-UC/IBD-U 58.8% vs. EO-UC/IBD-U 58.3%) (Supplementary Table S3). Among them, the proportion of UC/IBD-U patients under TNF-α inhibitors was similar in both cohorts (VEO-UC/IBD-U 60% vs. EO-UC/IBD-U 57.1%) whereas the proportion tended to be higher in EO-CD (75%) than VEO-CD (42.9%). All but one VEO-IBD patients who were not in SFCR (refractory disease) (*n *= 10) were under biological treatment, alone or in combination with steroids or IM. In contrast, only 40% of EO-IBD patients with refractory disease (*n *= 25) received TNF-α inhibitors (Supplementary Table S3). One VEO-CD patient underwent a subtotal colectomy 9 months after diagnosis because of a severe refractory Crohn's disease, resistant to anti-TNFα therapy. Two EO-CD patients underwent a segmental resection of the small bowel and an ileocaecal resection at 6- and 11-months post-diagnosis, respectively. No patient with UC or IBD-U had a surgery at the end of the first-year follow-up.

At the end of follow-up, the median BMI *z*-score was 0.46 (IQR = −0.6/1.6) in VEO-IBD patients vs. 0.2 (IQR = −0.7/1.2) in the EO-IBD group. SFCR was observed with similar frequency in both groups: in CD, 81.8% vs. 81.4% of patients were in SFRC in VEO-CD and EO-CD group respectively; in UC/IBD-U, 64.6% vs. 75% of patients were in SFCR in VEO-UC/IBD-U vs. EO-UC/IBD-U group. Among patients in SFCR at the end of follow-up, biologic therapies were used in 70% of VEO-IBD patients vs. 77.3% in the EO-IBD group (VEO-CD 66.7%; EO-CD 88.6%; VEO-UC/IBD-U 72.7%; EO-UC/IBD-U 55.5%). Three VEO-IBD (12.5% of SFCR) and 2 EO-IBD (3.5% of SFCR) patients were in SFCR without any treatment (Table 4). As shown in Table 4, clinical remission was confirmed with a fecal calprotectin less than 200 or a normal endoscopy in 71% of CD patients and 73% of UC/IBD-U patients.

**Table 4:** Treatment and outcomes at the end of follow-up.

		CD		UC and IBD-U	
		VEO	EO	VEO	EO
		*n *= 11 (%)	*n *= 43 (%)	*n *= 17 (%)	*n *= 24 (%)
**Steroid Free Clinical Remission (SFCR): Total**		**9** **(81.8)**	**35** **(81.4)**	**11** (**64.6)**	**18** **(75)**
**SFCR** Fecal Calprotectin (FC) <200	Anti-TNF-α alone	2 (18.2)	17 (39.5)	1 (5.9)	6 (25)
	Anti-TNF-α + IM	1 (9.1)	3 (7.0)	1 (5.9)	1 (4.2)
	Ustekinumab	0	1 (2.3)	0	0
	Vedolizumab	0	0	1 (5.9)	0
	IM alone	0	1 (2.3)	1 (5.9)	4 (16.7)
	Mesalamine	0	0	0	1 (4.2)
	Sulfasalazine	1 (9.1)	0	0	1 (4.2)
	No treatment	0	0	1 (5.9)	0
**SFCR** Mucosal healing (normal endoscopies)	Anti-TNF-α alone	1 (9.1)	1 (2.3)	0	1 (4.2)
	Anti-TNF-α + IM	0	1 (2.3)	0	0
	Vedolizumab	0	0	1 (5.9)	0
	IM alone	0	1 (2.3)	1 (5.9)	0
	No treatment	1 (9.1)	0	0	0
**SFCR** No assessment of FC or mucosal healing available	Anti-TNF-α alone	1 (9.1)	6 (14)	3 (17.6)	2 (8.4)
	Anti-TNF-α + IM	1 (9.1)	2 (4.6)	1 (5.9)	0
	Mesalamine	0		0	2 (8.4)
	No treatment	1 (9.1)	2 (4.6)	0	0
**Remission on Steroids**		**0**	**1** **(2.3)**	**1** (**5.9)**	**1** **(4.2)**
**Absence of remission: Total**		**1** **(9.1)**	**6** **(14.0)**	**2** (**11.8)**	**3** **(12.4)**
Absence of remission	Anti-TNF-α alone	0	2 (4.6)	0	2 (8.4)
	Anti-TNF-α + IM	1 (9.1)	1 (2.3)	0	0
	Ustekinumab	0	1 (2.3)	0	0
	IM alone	0	1 (2.3)	0	0
	Steroids + IM	0	1 (2.3)	0	0
	Mesalamine	0	0	0	1 (4.2)
	Sulfasalazine	0	0	1 (5.9)	0
	No treatment	0	0	1 (5.9)	0
**Post-colectomy**	**No medical treatment**	**0**	**0**	**2** **(11.8)**	**1** **(4.2)**
**Lost to follow-up**	** **	**1** **(9.1)**	**1** (**2.3)**	**1** **(5.9)**	**1** **(4.2)**

In total, during the follow-up, 71.4% VEO-IBD and 71.6% EO-IBD patients were treated by biologic therapies. The median time to first biologic treatment after diagnosis was 3.5 and 1.5 months in the VEO-IBD and EO-IBD groups, respectively (Table 4). At the end of follow-up, 3 patients (10.7% of the VEO-IBD cohort) had undergone a colectomy, two of whom were in clinical remission without medical therapy. Four patients required surgery in the EO-IBD cohort (two ileocecal resections, one segmental resection of the small bowel and one colectomy).

## Discussion

In our cohort, patients less than 10 years old at diagnosis (defined as A1a according to the Paris IBD classification) represented 17.3% of the whole pediatric cohort, with VEO-IBD accounting for 5.1% and EO-IBD for 12.2%. This is in line with most published cohorts where VEO-IBD consisted of 3%–5.8% of patients under 18-year-old (8–10). The frequency of CD was higher in older patients, whereas VEO-IBD patients were more frequently diagnosed with IBD-U, which also compared with the published cohorts (8, 10, 12, 13).

Upper GI endoscopy, performed in 100% of CD patients, and radiology performed in 98.1% of CD patients, revealed that L4 disease was significantly less frequent in VEO-IBD (54.5%) compared to EO-IBD (81.4%) (*p* < 0.009). This difference is by the trend reported in the literature where L4 phenotype was described in 2.3%–43.8% and 9.2%–59.9% of VEO-IBD and EO-IBD, respectively (10, 11). No L4b disease was observed in VEO-IBD, whereas 18.7% of EO-IBD had a jejunal or proximal ileal involvement. Dhaliwal et al. reported that 27% of a whole pediatric cohort had L4b disease without specifying the rate for VEO-IBD patients (8). In total, higher rates of upper GI involvement (L4) were observed in our study. This could be due to more systematic use of upper GI endoscopy or radiologic examination in our more recent cohort. Indeed, all patients but three (96.8%) had imaging examinations in our cohort, including a MRE in 33.3% and 92.5% of VEO-IBD and EO-IBD patients, respectively. MRE in very young patients was not obtained as frequently as in EO-IBD because it required general anesthesia in this age group. In contrast, children of 5 years of age and older can usually perform MRE without sedation. When a MRE could not be performed, the small bowel was assessed by an abdo-US which has been shown to have a 90% sensitivity to detect disease in adult patients (21). Also, one-third of VEO-IBD and 4 EO-IBD patients underwent a SBFT to complete the radiologic investigations. Therefore, the higher frequency of L3 and L4 phenotypes observed in the EO-IBD group is not a consequence of under-evaluating the VEO-IBD group.

Consistent with this, histological findings in the upper digestive tract were more frequent in the EO-IBD than VEO-IBD group, in both CD and UC/IBD-U patients. Overall, normal upper GI tract biopsies were found in 42.8% of the VEO-IBD patients compared to only 16.4% in the EO-IBD cohort. In the lower digestive tract, no clear difference was seen in terms of disease activity, defined by neutrophilic infiltration between both groups, as previously reported by Conrad et al. (14). Also, granulomas were found in a similar high proportion (∼65%) in both cohorts. Other investigators have reported a comparable prevalence of granulomas across ages in pediatric cohorts, however, with a wide range of frequencies depending on the studies (30%–51%) (8, 14). A histological signature of VEO-IBD has been proposed by Conrad et al., including a higher frequency of diffuse eosinophilic infiltration of the mucosa with involvement of the crypt, lamina propria, and surface epithelium, as well as a higher frequency of apoptosis (14). In our cohort, we did not report higher frequencies of eosinophils or apoptotic bodies in the VEO-IBD compared to the EO-IBD group. We questioned whether the discrepancies between our results and Conrad's results were due to the absence of monogenic VEO-IBD in our cohort. However, Conrad et al. reported no difference between the monogenic and non-monogenic VEO-IBD regarding the frequencies of the histological features observed. Contrary to most studies reporting specific features of VEO-IBD cohorts compared to all patients above 6 years at diagnosis, we aimed to compare very young patients to young patients and did not include patients older than 10 years at diagnosis. Therefore, we can hypothesize that the differences observed in Conrad's study between VEO-IBD and older patients are driven by patients older than 10-year-old who represent the majority of the pediatric cohorts.

In our cohort, corticosteroids were used as induction treatment with a higher frequency in VEO-IBD than EO-IBD. This contrasts with other groups reporting an equal use of steroids as first-line therapy across ages (8, 10). At 1-year post-diagnosis, clinical remission was achieved in the same proportion of patients in each group (∼60%), but a lower rate of VEO-IBD patients was under biologic treatment. More than half of VEO-IBD patients were treated with TNF-α inhibitors at 1-year follow-up in our cohort, contrasting with only 15%–18% reported previously (12, 22). During a median follow-up of 56 months, we reported 71.4% of VEO-IBD and 74.6% of EO-IBD patients treated with biologic therapy. The similar rate of biologics use in both cohorts was consistent with several other research groups (10, 11). Combined with the fact that the SFCR rate at the end of follow-up was similar in both groups (85%), we conclude that the outcome was comparable in both cohorts.

In total, consistent with the literature, VEO-IBD patients were more frequently diagnosed with IBD-U, whereas EO-IBD patients were more likely to have CD. The VEO-CD group had a higher prevalence of isolated colonic CD, whereas ileocolonic disease predominated in EO-CD patients. In UC and IBD-U, the extent of the disease was similar in both groups, mainly presenting with pancolitis. Interestingly, the prevalence of lesions (including macroscopic and histological features) in the upper gastrointestinal tract was higher in the EO-IBD than in the VEO-IBD cohort in all disease subtypes. Also, we did not find a differential histological signature (neutrophilic and eosinophilic infiltration, apoptotic bodies, and granulomas) in the ileum and colon between VEO-IBD and EO-IBD patients. Finally, the requirement for biologic therapies during follow-up was similar in both groups. In both cohorts, about 40% of patients were not in SFCR at 12 months post-diagnosis despite an early use of biologics therapies. However, at the end of a median follow-up of 56 months, 85% of VEO-IBD and EO-IBD patients eventually achieved SFCR, with an overall similar disease course in both groups.

## Data Availability

The original contributions presented in the study are included in the article/**Supplementary Material**, further inquiries can be directed to the corresponding author.
