# Supplementary material for: Phenotype and outcomes of very early onset and early onset inflammatory bowel diseases in a Montreal pediatric cohort
Source: Front Pediatr. 2023 Apr 4;11:1157025. doi: 10.3389/fped.2023.1157025 (PMC10110991; doi:10.3389/fped.2023.1157025)
Supplement: Supplementary file 1 [file Datasheet1.docx]

**Supplementary tables**

**Table S1.** Patient genetic characteristics

|  | VEO-IBD | EO-IBD |
| --- | --- | --- |
| Number of patients tested  (Commercial IBD gene targeted panels) | *n* = 12 (42.9%) | *n* = 5 (7.5%) |
| Heterozygous carrier of VUS  (Variant of unknown significance) | *n* = 6  *ADAM17*  *TCC7A*  *HPS6*  *LIG4*  *DOCK2*  *DOCK8*  *IL10RA* | *n* = 1  *NCF2******** |

******* Carrier of two heterozygous variants of the NCF2 gene, one likely pathogenic and the other one of unknown significance. However, the patient's neutrophil oxidation test was normal, excluding a diagnosis of chronic granulomatous disease.

**Table S2.** Radiologic data

|  | **VEO-IBD** n (%) | **EO-IBD**  n (%) |
| --- | --- | --- |
| **Imaging Examinations** |  |  |
| Abdominal Ultrasound (US)  Magnetic Resonance Enterography (MRE)  Neither MRE nor Abdominal US  Small Bowel Follow Through  Abdominal CT-Scan | 26 (96.3)  9 (33.3)  1 (3.7)  9 (33.3)  1 (3.7) | 50 (74.6)  62 (92.5)  2 (3.0)  4 (6.0)  1 (1.5) |
| **Intervals** |  |  |
| Average Interval Between Diagnosis and US, days (SD)  Average Interval Between Diagnosis and MRE, days (SD) | 9.0 (71.2)  7.3 (5.9) | 7.0 (103.3)  34.7 (106.8) |
| **Ileal lesions on MRE** |  |  |
| Short < 15cm  Medium 16-30cm  Long > 30cm  Undefined Size of Lesions | 0 (0.0)  1 (8.3)  0 (0.0)  0 (0.0) | 11 (17.7)  3 (4.8)  3 (4.8)  8 (12.9) |

Radiologic data were available at diagnosis for all patients but one in the VEO-IBD group.

**Table S3.** Treatment and Outcomes at one year (± 2 months) post-diagnosis

|  | CD | | UC and IBD-U | |
| --- | --- | --- | --- | --- |
|  | VEO  *n*=11 (%) | EO *n*=43 (%) | VEO  *n*=17 (%) | EO *n*=24 (%) |
| **Steroid Free Clinical Remission (SFCR): Total** | **7 (63.6)** | **28 (65.1)** | **10 (58.8)** | **14 (58.3)** |
| Anti-TNF-ꭤ alone | 2 (18.2) | 19 (44.3) | 3 (17.6) | 5 (20.8) |
| Anti-TNF-ꭤ + IM | 1 (9.1) | 2 (4.6) | 3 (17.6) | 3 (12.5) |
| IM alone | 3 (27.3) | 6 (14.0) | 1 (5.9) | 2 (8.3) |
| Sulfasalazine | 1 (9.1) | 0 | 1 (5.9) | 1 (4.2) |
| Mesalamine | 0 | 1 (2.3) | 0 | 3 (12.5) |
| No treatment | 0 | 0 | 2 (11.8) | 0 |
| **Absence of remission: Total** | **3 (27.3)** | **15 (34.9)** | **7 (41.2)** | **10 (41.7)** |
| Anti-TNF-ꭤ alone | 1 (9.1) | 7 (16.3) | 1 (5.9) | 3 (12.5) |
| Anti-TNF-ꭤ + IM | 2 (18.2) | 0 | 1 (5.9) | 0 |
| Anti-TNF-ꭤ + IM + steroids | 0 | 0 | 2 (11.8) | 0 |
| Anti-TNF-ꭤ + steroids | 0 | 0 | 1 (5.9) | 0 |
| vedolizumab + IM + steroids | 0 | 0 | 1 (5.9) | 0 |
| IM | 0 | 1 (2.3) | 0 | 3 (12.5) |
| IM + steroids | 0 | 0 | 1 (5.9) | 0 |
| mesalamine | 0 | 2 (4.6) | 0 | 4 (16.7) |
| Steroids alone | 0 | 3 (7.0) | 0 | 0 |
| No treatment | 0 | 2 (4.6) | 0 | 0 |
| **Lost of follow-up** | **1 (9.1)** | **0** | **0** | **0** |
